# Teriparatide Therapy as an Adjuvant for Tissue Engineering and Integration of Biomaterials

**DOI:** 10.3390/ma4061117

**Published:** 2011-06-15

**Authors:** Robinder S. Dhillon, Edward M. Schwarz

**Affiliations:** 1 The Center for Musculoskeletal Research, University of Rochester, Rochester, NY 14623, USA; E-Mail: robin_dhillon@urmc.rochester.edu; 2 Department of Orthopaedics, University of Rochester, Rochester, NY 14623, USA

**Keywords:** Parathyroid Hormone (PTH), teriparatide, osseointegration, bio-integration, fracture healing, non-union, allograft repair, biomaterials

## Abstract

Critically sized large bone defects commonly result from trauma, radical tumor resections or infections. Currently, massive allografting remain as the clinical standard to treat these critical defects. Unfortunately, allograft healing is limited by the lack of osteogenesis and bio-integration of the graft to the host bone. Based on its widely studied anabolic effects on the bone, we have proposed that teriparatide [recombinant parathyroid hormone (PTH_1–34_)] could be an effective adjuvant for massive allograft healing. In support of this theory, here we review studies that have demonstrated that intermittent PTH_1–34_ treatment enhances and accelerates the skeletal repair process via a number of mechanisms including: effects on mesenchymal stem cells (MSC), angiogenesis, chondrogenesis, bone formation and remodeling. We also review the current literature on the effects of PTH_1–34_ therapy on bone healing, and discuss this drug’s long term potential as an adjuvant for endogenous tissue engineering.

## 1. Introduction

While bone has regenerative capabilities that enable self-repair of fractures, this is not possible in extreme situations that involve massive bone loss. Such bone defects termed ‘critically sized defects’ (>2 cm size) are often a result of infection, trauma or radical cancer surgery. The global burden of these critical defects is enormous as approximately 1.6 million bone grafts are performed each year. From a total of over 500,000 yearly bone graft procedures performed in the US alone, over 175,000 (35%) are bone allografts [1]. Furthermore, oral cancer is a major reason for mandibulectomy and maxillectomy; an estimated 34,000 Americans and over 640,000 people worldwide will be diagnosed with oral cancer this year [2]. Interestingly, more than 70% of all military-related extremity injuries require bone grafts for critical sized defect repair [3]. Autologous bone transplantation is the gold standard treatment for bone loss reconstruction and over 275,000 autograft procedures are performed yearly in the US [4]. Also of note is the variable degree of impaired healing in 5% to 10% of the 7.9 millionfractures occurring annually in the United States [5]. 

## 2. The ‘Need’ in Critical Sized Defects

Unfortunately, autologous bone is not always an option and is not devoid of complications such as pain, infection, injury to surrounding structures and its harvest invariably requires the mutilation of another bone. Often, the repair achieved using this technique is not efficient. The closest alternative option available in this scenario is ‘structural’ allograft. Although allografts are widely available, they are prone to failure due to their limited ‘osseointegration’ with the host that remains a major challenge. Unfortunately, allografts have a failure rate of 60% at 10 years [6]. This has been attributed to limited new bone formation and neo-vascularization, propagating micro-fractures and fibrotic non-unions Thus, it is clear that if a non-autologous bone graft tissue engineering solution of critical defect reconstruction is to be realized, a novel adjuvant therapy that can facilitate osseointegration of the construct will be necessary for this unmet clinical need. In addition, this adjuvant therapy must be able to harness the inflammation associated with the foreign body reaction that has doomed all of the other approaches that have been attempted previously.

## 3. The ‘Need’ in Impaired Fracture Healing 

The delayed union and non-union of fractures are the reasons for pain and disability in a large subset of fracture patients. There could be a number of factors affecting the optimal fracture repair process: (a) patient related factors include advanced age, diabetes, osteoporosis, corticosteroid treatment, *etc.*; (b) injury related factors include massive injury, open and complex fractures and; (c) surgical treatment related factors include improper fixation, incorrect or short immobilization, infection. Currently, most of the patients with delayed and non-union, unfortunately, have to undergo surgical intervention. There is still a dearth of treatment(s) that could effectively enhance repair in the face of these sub-optimal conditions. Such a treatment would have a huge social and economic impact. 

## 4. PTH Physiology

Parathyroid hormone (PTH) is an 84 amino-acid peptide (1–84) hormone that is a major systemic regulator of calcium homeostasis along with calcitonin [7,8,9]. It is released from the parathyroid gland, and its secretion controlled chiefly by serum [Ca^2+^] through negative feedback [10]. In response to hypocalcemia, PTH release increases serum calcium concentration by promoting osteoclast-mediated bone resorption, calcium reabsorption in the kidneys, and intestinal absorption of calcium. Therefore, continuous exposure to PTH, as in hyperparathyroidism, leads to hypercalcemia and a net decrease in bone volume, which is referred to as its ‘catabolic effect’. In contrast, intermittent (once daily) exogenous PTH administration has an anabolic effect on bone [11]. 

## 5. Teriparatide

Teriparatide is the biologically active, recombinant N-terminal 1–34 amino acid polypeptide of naturally occurring human PTH. Teriparatide (PTH_1–34_), by virtue of its rather unique mechanism of action on bone, has been FDA approved as the only anabolic therapy for treating patients with osteoporosis who are at high risk for future fracture [12]. In osteoporosis, teriparatide works as an anabolic agent enhancing bone formation throughout the body skeleton by relatively enhancing osteoblast-derived bone formation in comparison to osteoclast-derived bone resorption, with a resultant net increase in bone mass [13,14]. Teriparatide has since found clinical application in treatment of both post-menopausal and glucocorticoids induced osteoporosis [15,16]. Considering that callus formation and mineralization are critical steps to fracture and allograft healing, there is a strong rationale for PTH_1–34_ therapy to accelerate and enhance skeletal repair. 

Additionally, evidence that teriparatide enhances chondrogenesis has generated interest in its scope for articular cartilage repair [13,17]. Research is underway to understand the effects teriparatide may have on mesenchymal stem cells, and studying other effects that have been reported incidentally in patients using the drug for osteoporosis, including the healing of fracture non-unions and decreased incidence of back pain. Here we review the current animal and human research on the uses of teriparatide in musculoskeletal diseases beyond osteoporosis, with a focus on ‘bio-integration’. We also address the critical issues that must be resolved to achieve the desired clinical application.

### Continuous *vs*. Intermittent Debate

It is now widely accepted that intermittent PTH is anabolic, whereas continuous PTH is catabolic [18,19]. Although several mechanisms have been postulated for this observation, the exact mechanisms for these differential effects remains incompletely understood. However, some of the anabolic effects of intermittent PTH have been attributed to: proliferation and differentiation of osteoprogenitor cells in bone marrow [20], actions on post-mitotic preosteoblasts on bone [21], prevention of osteoblast apoptosis [22], and reduced expression of sclerostin by osteocytes [23]. In contrast, continuous PTH infusion leads to persistently and markedly enhanced bone resorption [24] and suppression of bone formation from discordant effects on 1,25(OH)_2_ vitamin D [25], increased RANKL and decrease OPG expression [26]. 

## 6. Current Approaches to Revitalize Bone Allografts

**Figure 1:**
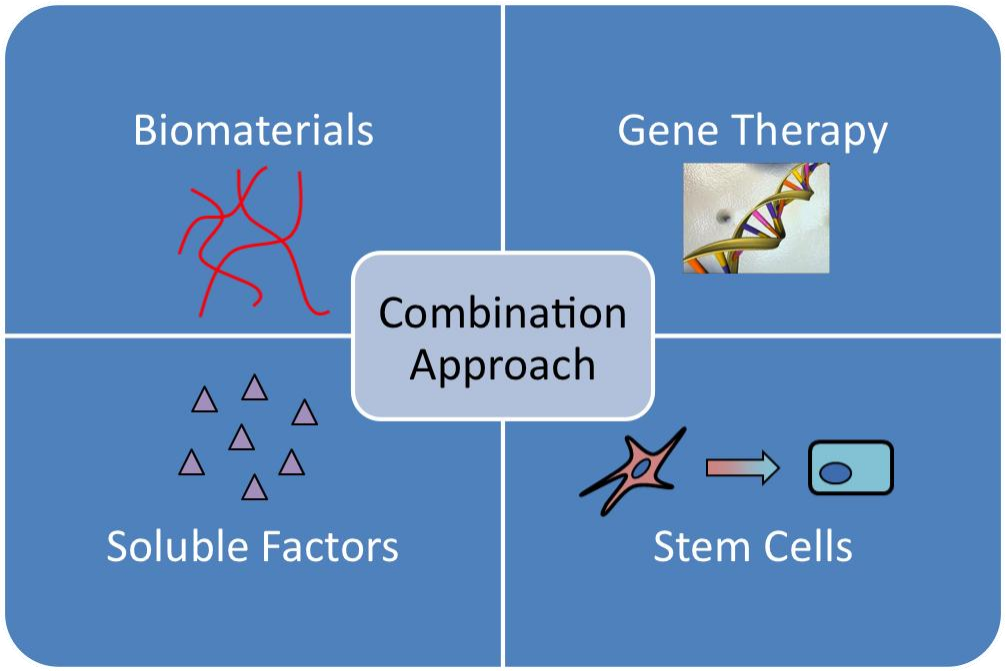
Current Strategies to Revitalize Bone Allografts.

The use of locally implanted or injected growth factors has received much attention lately, but the systemic treatments for the augmentation of bone repair, especially in situations where the bone repair may be absent or delayed, are now under investigation [27,28,29]. The major approaches to address the problem of graft failure are the modulation of the hosts’ internal environment to become more receptive and conductive for osseointegration and external adjuvant therapy of the allograft to promote osseointegration. Four broad strategies that have been pioneered for revitalizing of the allografts are gene therapy, stem cell therapy, usage of biomaterials and soluble factors (Figure 1).

Most studies in which the goal is to overcome the loss of large bone segments involve administration of cells with osteogenic potential or osteogenic growth factors [30,31,32]. Intermittent teriparatide treatment of MSCs *in vitro* led to increased formation of total and ALP positive CFU (colony forming units) [20]. Teriparatide may have a role in MSC lineage determination, as PTH treatment decreases their differentiation into adipocytes [33], and enhances their osteogenic differentiation [34,35]. However, PTH does not stimulate MSC proliferation [35]. 

MSCs have been widely recognized as a potential therapy for bone regeneration. However, most studies have concluded that use of MSCs without an osteogenic stimulus has little effect on bone formation. Thus, MSCs are typically used in combination with gene therapy, soluble factors and biomaterials. Unfortunately, serious safety concerns continue to limit the clinical development of human gene therapy, and we have shown that while MSC can be used as the delivery vehicle [36,37,38,39], two practical issues have impeded its clinical development. The first pertains to the source of MSCs. If they are autologous, then they must be cultured for weeks in advance, to generate enough cells for the surgery, which is cost (money, time, labor) prohibitive as demonstrated by the Carticell^TM^ experience. If they are from an allogenic bank there are concerns regarding immunogenicity and quality control. Nevertheless, preclinical studies have demonstrated several remarkable results. Studies of *ex vivo* regional gene therapy with bone marrow cells over expressing bone morphogenetic protein-2 (BMP-2) have demonstrated efficacy in healing critical sized bone defects. This “same day” strategy offers a solution to restrictions associated with the culture expansion process required in the traditional *ex vivo* approach [40]. Taking it a step further, Aslan H. *et al*. demonstrated that immunoisolated noncultured CD105-positive [CD105(+)] hMSCs are multipotent *in vitro* and exhibit the capacity to form bone *in vivo* [41]. Virk M. *et al*. also demonstrated *in vivo* bone formation using hMSCs nonvirally transfected with the human bone morphogenetic protein-2 (hBMP-2) or -9 (hBMP-9) [42]. Given these major advances towards clinical translation, many in the field are optimistic that if concerns about the safety and cost-effectiveness can be abated, genetically modified stem cells hold great promise for regenerative bone therapy. 

In the soluble factors category, two treatments have shown promise, BMP and Teriparatide. The recombinant human osteogenic growth factors (for example, BMP-2 and BMP-7) have been introduced into clinical practice and have shown great promise [29,43]. However, due to their high cost and limited applicability, the use of these proteins is optimal only for small bone lesions. Teriparatide has been investigated in animal models and in patients as a potential agent to enhance fracture repair and revitalize allografts. Recently our research group has made remarkable discoveries suggesting that clinically relevant doses of teriparatide may be this long sought after adjuvant therapy that begs further investigation. 

## 7. Advantages of Teriparatide Therapy in Treating Problematic Fractures

Since available adjuvant therapies against impaired bone healing are limited, PTH_1–34_ therapy could be a reliable and safe treatment option for these conditions. Although local therapy using osteogenic factors such as bone morphogenetic protein (BMP-2 and BMP-7) is another attractive option as an adjuvant treatment for skeletal repair based on its successful use in spinal fusion surgery experience [29], PTH_1–34_ has some advantages over the local growth factor therapy. Local growth factor therapy requires surgical implantation with a carrier material at the surgical site, and is only effective for a few days. In contrast, PTH_1–34_ therapy can be pragmatic to any type of skeletal injury, and has inherent advantages of being a non-operative option, control over the administration and dosage and duration. As commonly observed, most fractures and fracture complications occur in elderly or in disease states. Consistent with this clinical observation, Jilka *et al.* recently reported that aged mice have increased oxidative stress and decreased bone anabolism that can be overcome with teriparatide treatment [44]. Thus, a potential benefit of PTH_1–34_ therapy is enhanced healing in the patients who suffer from delayed or non-unions.

## 8. The Basic Science behind Teriparatide

Andreassen *et al.* in 1999, was the first to report the efficacy of intermittent PTH_1–34_ therapy on rat tibial fracture healing [45]. Since then a number of studies have shown that PTH_1–34_ enhances bone repair regardless of the skeletal site [13]. These studies suggest that PTH_1–34_ plays a role not only in bone remodeling, but also in modulation of osteogenesis and chondrogenesis during skeletal repair, thereby leading to dramatic effects on bone healing. We have now learned from various skeletal repair models that PTH_1–34_ exerts its action through multiple intricate mechanisms. Cellular mechanisms in PTH_1–34_ driven bone repair include, but are not limited to, proliferation and differentiation of MSC, chondroprogenitors and osteoprogenitors. It also affects chondrocyte maturation, production of bone matrix proteins, and osteoclastic remodeling. 

The most prominent effects of PTH_1–34_ on bone repair are on proliferation and differentiation of MSC, chondroprogenitors and osteoprogenitor cells at the fracture site in a rat fracture model with a resultant increased callus formation during fracture healing process [46,47]. *In vitro* studies implicate the induction of *osterix* and *runx2* expression by PTH_1–34_ for enhanced MSC differentiation into osteoblasts [48]. Additional *in vivo* fracture studies demonstrate increased expression of osterix and other osteoblastic marker genes (*osteocalcin* and *Type 1 collagen)* at the fracture site with daily 40 μg/kg PTH_1–34_ treatment [34,49]. Thus, it can be concluded that systemic PTH_1–34_ has direct effects on MSC and osteoblast gene expression. Additionally, increased cartilage in the fracture callus is prominent finding of PTH_1–34_ therapy during fracture healing that occurs through interplay between delayed chondrocyte hypertrophy and remodeling. 

In terms of cellular targets, PTH_1–34_ is known to have direct effects on MSC, chondrocytes and osteoblasts during endochondral bone healing [50]. Kakar *et al.* showed that PTH_1–34_ preferentially enhanced chondrogenesis over osteogenesis in their mouse closed femoral fracture model [51]. This enhanced chondrogenesis leads to increased cartilaginous callus formation in the early phase of fracture repair; subsequently the PTH_1–34_ enhances chondrocyte maturation and mineralization in the fracture callus, as evidenced by both an earlier peak in *Sox9* expression and the corresponding earlier induction of *type X collagen*. Furthermore, the induction of the Indian hedge-hog (IHH) and wnt/β-catenin pathways has also been implicated in this process [17]. PTH_1–34_ also has significant effects on membranous ossification as established in bone defect and bone-chamber models [52,53,54].

Komatsubara *et al*. investigated the effects of PTH_1–34_ therapy on the later phase of fracture healing, focusing on callus remodeling and geometrical changes in a rat femoral osteotomy model [55]. PTH_1–34_ was found to accelerate the remodeling of woven bone to lamellar bone in the callus. The group confirmed their findings in a primate study later, concluding that PTH_1–34_ accelerates the remodeling process of fracture callus by accelerated mineralization and the shrinkage of the fracture callus size thereby restoring the form and function of the bone [56].

Bone formation is a dynamic process *i.e*., it is a net output of combined osteoblast and osteoclast function. PTH_1–34_ is known to increase the bone formation rate via direct stimulation of osteoblast function, inhibition of apoptosis that extends the life of osteoblasts and sustained osteoblast activity during the remodeling period [22]. PTH action on osteoblasts leads to changes in the synthesis and/or activity of several proteins, including osteoclast differentiating factor, also known as TRANCE, RANKL, or OPG (osteoprotegerin) ligand [57,58]. While osteoclasts too, are considered to play a vital role in callus remodeling, the research evidence on PTH_1–34_ action on osteoclasts is unclear. Several studies reported that PTH_1-34_ increases osteoclast activity during fracture healing [46,55], some others showed that osteoclast density within the fracture callus does not change with PTH_1–34_ treatment [59], whereas one study reported that PTH_1–34_ down-regulates osteoclast activity, as measured by serum TRAP5b levels [52]. Most studies in osteoporosis treatment, however, attribute the anabolic actions of PTH to increased osteoblast activity [60,61,62]. The resorptive effect of continuous PTH is predominantly due to stimulation of osteoclast differentiation and activity via effects on osteoblast cytokine production. The precise modulation of the function of osteoclasts with PTH is still not fully understood. It has been suggested that for a sustained anabolic effect of teriparatide, the resorptive action is essential [62].

## 9. The Prospect of PTH Therapy in Osseointegration—Experience from Animal Studies

The most important part of biomechanical allograft healing is the osseous integration of the graft to the host bone. Recently, we developed a micro-CT and CT based algorithm, termed the Union Ratio, as a non-destructive quantitative predictor of the torsional biomechanics of the allografted bone [63]. In these studies, we compared the union ratio (union between host callus and graft) of live autografts to devitalized allografts implanted into the mid-diaphysis of mouse femurs for 6 and 9 wk. The allograft union ratio was 0.105+/−0.023 at 6 week but increased to 0.224+/−0.029 at 9 weeks (p < 0.05). As a single variable, the union ratio correlated significantly with ultimate torque (R (2) = 0.58) and torsional rigidity (R (2) = 0.51) of the allografts. Multivariable regression analyses of allografts that included the union ratio, the graft bone volume, the maximum and minimum polar moment of inertia, and their first-order interaction terms with the union ratio as independent variables resulted in significant correlations with the ultimate torque and torsional rigidity (adjusted R (2) = 0.80 and 0.89, respectively). These results suggest that, unlike live autografts, the union between the devitalized allograft and host contributes significantly to the strength of grafted bone. The union ratio has important clinical implications as a novel biometric for noninvasive assessment of functional strength and failure risk.

Subsequently, we investigated the effects of daily systemic injections of 40 μg/kg teriparatide on the allograft healing in a mouse model [25]. The femurs were evaluated at 4 and 6 weeks using microCT, histology and torsion testing. Teriparatide induced prolonged cartilage formation at the graft-host junction at 4 weeks, which led to enhanced trabeculated bone callus formation and remarkable graft-host integration at 6 weeks. Also observed was a significant 2-fold increase in normalized callus volume (1.04 ± 0.3 *vs.* 0.54 ± 0.14 mm³/mm; p < 0.005), and Union Ratio (0.28 ± 0.07 *vs.* 0.13 ± 0.09; p < 0.005), compared to saline treated controls at 6-weeks. Teriparatide treatment also significantly increased the torsional rigidity (1175 ± 311 *vs.* 585 ± 408 N.mm²) and yield torque (10.5 ± 4.2 *vs.* 6.8 ± 5.5 N.mm) compared to controls. Interestingly, the Union Ratio correlated significantly with the yield torque and torsional rigidity (R² = 0.59 and R² = 0.77, p < 0.001, respectively). These results illustrate the remarkable potential of teriparatide as an adjuvant therapy for allograft repair in a mouse model of massive femoral defect reconstruction, and warrant further investigation in a larger animal model at longer time intervals to justify future clinical trials for PTH therapy in limb sparing reconstructive procedures.

## 10. The Prospect of PTH Therapy in Human Skeletal Repair—Evidence So Far

Harping on the success of PTH_1–34_ in osteoporosis and evidence from the animal experiments, PTH_1–34_ has found an off label application in treating delayed unions and non-unions [64,65,66]. As one would imagine it is not possible to test the biomechanical properties of osseointegration in humans in comparison to animal models. There are published case reports that study the union ratio which is a surrogate marker of the strength of repair [63]. One of the earliest case reports was the treatment of pelvic fracture, where 5 weeks of PTH_1–34_ treatment led to 20% increase in the callus volume [67]. Another report describes the successful treatment of a radiologically confirmed non-union of tibia 4 months after initial injury, managed conservatively with 8 weeks of daily PTH_1–34_ S/C injection treatment in an otherwise healthy adult male. Also upon CT scan measurements they found 278% increase in the union (contact) area before and after treatment [64]. This interesting and rather striking observation sparked a whole host of animal research and also experimental/off label clinical usage internationally. Since then case studies have reported the use of PTH_1–34_ for successfully treating sternal non-union [66] and humeral non-union [63]. Resmini *et al.* described a case of 79-year old woman taking teriparatide for her osteoporosis who demonstrated significant healing of her left proximal humerus fracture after only 25 days [68]. Rubery and Bukata described a case series of 3 patients with non-unions of Type 3 odontoid fractures, healed successfully with PTH_1–34_ treatment [65]. Peichl *et al.* reported on a series of five elderly patients treated with PTH (1–84) with pubic rami fractures, all of whom demonstrated pain resolution and healing by 8 weeks compared with the normal 12–16 weeks typically seen [69]. Recently another interesting clinical case study depicts the successful non-surgical management of the aseptic loosening of a twice revised hip prosthesis with 8 months of daily teriparatide therapy [70]. In this case the PTH_1–34_ treatment not only relieved the pain of the patient but also improved his peri-prosthetic BMD (Bone Mineral Density) and WOMAC (Western Ontario and McMaster Universities Osteoarthritis Index) scores. This sets the stage for a whole new foray of clinical application of PTH_1–34_.

Although the clinical case reports of off-label use of PTH_1–34_ to heal non-unions support the potential for its application as an adjuvant for impaired bone healing, but the clinical evidence for widespread teriparatide use still remains missing as the case studies and case series only provide anecdotal evidence (level-4 evidence). Surprisingly there is only one published randomized, double blind, placebo-controlled clinical trial testing the effect of PTH_1–34_ in treating distal radial fractures. Aspenberg *et al.* carried out this trial on a group of 102 postmenopausal women that sustained a distal radius fracture [71,72]. The study failed to prove a statistically significant difference in time to cortical bridging (radiological fracture healing) in the high dose teriparatide treatment group (40 μg/day). Interestingly, this study did show that 20 μg daily PTH_1–34_ treatment, shortened the time to radiographic healing from 9.1 to 7.4 weeks *versus* placebo group. The clinical relevance of this observation yet remains unclear. The unequivocal results of this trial highlight the pressing need for future clinical trials to translate the basic science experience into clinical practice. 

## 11. Biochemical Markers of PTH_1–34_ Therapy

Biochemical markers of bone turnover may be useful aids for managing patients with osteoporosis along with a secondary objective measure of PTH action. A phase 3, multicenter, double blinded, placebo controlled trial of 207 Japanese patients at high risk of fracture was conducted to assess the effects of teriparatide 20 μg/day on BMD, serum markers of bone turnover, and determine its safety profile [73]. Bone turnover markers including procollagen type I N-terminal propeptide (PINP), bone-specific alkaline phosphatase (bone ALP) and type I collagen cross-linked C-telopeptide (CTX) were studied against objective measurements of lumbar spine, femoral neck, and total hip BMD at baseline, 3, 6, and 12 months. Observed increase in PINP at 1 month correlated best with the increase in lumbar spine BMD at 12 months (P < 0.01). The teriparatide treatment caused an increase in PINP >10 μg/L at either 1 or 3 months and an increase in lumbar spine BMD >/=3% at 12 months, this confirmed a strong relationship between early change in PINP and later change in lumbar spine BMD during teriparatide therapy. Hence, PINP monitoring may be a useful aid in the management of patients with osteoporosis during teriparatide treatment. These results could be further studied and possibly applied to monitor patients on teriparatide for any indication. Once-daily injections of teriparatide initially increase biochemical markers (PINP) of bone formation and resorption, but markers peak after 6–12 months and then decline despite continued treatment [74].

## 12. Unresolved Issues and Future Directions

There still exists a knowledge gap in our understanding of the indications for PTH_1–34_ use in musculoskeletal disease. As with any clinical treatment, benefit *vs.* risk must be weighed in and PTH_1–34_ use is no exception. Current FDA guidelines approve usage of PTH_1–34_ for up to a maximum of 2 years safely. Several months of PTH_1–34_ treatment is presumably required for skeletal repair. However, the potential risk of osteosarcoma needs to be carefully considered along with the high cost of the drug besides daily subcutaneous injections. 

### This Leads Us to Another Set of Questions Such As When to Start, What Dosage to Give, and for How Long Should the PTH_1–34_ Treatment Be Given?

Different clinical scenarios require cautious fine-tuning of the treatment. In trauma, it may be challenging to commence PTH_1–34_ treatment immediately, and it remains a mystery whether delayed PTH treatment is as effective as immediate treatment. Furthermore, there is no clinical information on how late PTH_1–34_ therapy can be initiated to achieve effective results on delayed and non-unions. PTH_1–34_ can be presumed to be effective for delayed-union whilst the bone healing process is still active at the fracture site, but the observed effects on non-unions, where bone formation is not active any more, remain a medical enigma. As shown in the distraction osteogenesis study, PTH_1–34_ therapy during the consolidation period is sufficient to show maximum effect on its healing [75], there may be an ideal treatment window for maximal benefit. Another critical question is the ideal dosage of PTH_1–34_ for the desired effect, *i.e*., whether osteoporosis treatment dose (20 μg/day) is appropriate for augmenting skeletal repair? There is the translational dilemma from rodent studies to human trials. Fracture healing studies in animal models, typically used much higher doses of PTH_1–34_ in range of 5–200 μg/kg/day, most commonly 40 μg/kg/day for observed anabolic effects on skeletal repair, this would correspond to non-physiologic doses (2.8 mg/day in a 70 kg adult) in humans, and it is thus uncertain whether these same effects would be seen in human at the much lower, approved physiologic dosage used currently. Hence there is the necessity for clinical trials in this area.

## 13. Conclusions

Evidence shows teriparatide to be an effective stimulator of bone remodeling for osteoporosis and shows assurance as a growth factor for fracture healing and bone fusion. One of its primary effects is the stimulation of the chondrocyte lineage of cells; this role looks very promising for treating osteoarthritis. Additional orthopedic uses, including the stimulation of healing for fracture non-unions, stimulation of bone ingrowth for porous stem orthopedic joint replacements and as a pharmacotherapy for management of loosened hip prosthesis yet remains to be fully investigated. The role of teriparatide in the directed stimulation of musculoskeletal and mesenchymal stem cells is being widely investigated and shows promise as a short-term use agent that may assist in the simultaneous healing of several musculoskeletal tissues after trauma or elective surgery. There is a need for further basic science and clinical research with this drug to understand its full potential and safe application for clinical use.
